# Antimony in soils of SW Poland—an overview of potentially enriched sites

**DOI:** 10.1007/s10661-019-7214-9

**Published:** 2019-01-15

**Authors:** Karolina Lewińska, Anna Karczewska

**Affiliations:** 1 Department of Soil Science and Remote Sensing of Soils, Adam Mickiewicz University in Poznań, ul. Krygowskiego 10, 61-680 Poznań, Poland; 2 Institute of Soil Science and Environmental Protection, Wrocław University of Environmental and Life Sciences, ul. Grunwaldzka 53, 50-357 Wrocław, Poland

**Keywords:** Antimony, Mining, Shooting range, Soil, Speciation, Geoaccumulation index

## Abstract

Great concern has been recently focused on antimony in the environment due to the potential risks posed by this metalloid to humans. In Poland, the concentrations of Sb in soils have not been well recognized. The aim of this study was to identify the sites in south-western Poland where soils are considerably enriched in Sb and to make a rough assessment of a related environmental risk. One hundred forty-four samples were collected from 20 Lower Silesian locations chosen as potentially enriched in Sb that included historical mining sites, tailings impoundments, close vicinities of operating copper smelters, and landfills as well as currently operating and historical shooting ranges. Total concentrations of Sb in soils were determined, and related pollution indices were calculated. Several locations were found where soils contain high concentrations of Sb, with a maximum that exceeded 5600 mg kg^−1^ Sb in the alluvial soil affected by historical mining. Sequential extraction showed a considerably high percentage of Sb extracted in potentially soluble fractions 1 (non-specifically sorbed) and 2 (specifically sorbed), particularly in the samples collected from shooting ranges and in some samples from historical mine areas. This result provides a strong premise for further risk- and remediation-oriented examination of soils in those sites. More detailed research is needed to determine a spatial extent of soil contamination in the sites identified as highly enriched in Sb. Contrary to these findings, soil material collected from copper tailings impoundments, surroundings of smelters, and landfills did not show any particular enrichment in Sb.

## Introduction

Concentrations of Sb in Polish soils have not been well recognized, and the only data available are those collected from the national monitoring of arable soils and grasslands, which revealed the Sb content in soils in the range 0.06–1.03 mg kg^−1^ (Pasieczna 2012). These data, compared with Sb concentrations in European topsoils, reported by Salminen et al. (2005), that range from 0.02 to 31.1 mg kg^−1^, and with the data provided by Kabata-Pendias (2011) for unpolluted soils 0.25–1.04 mg kg^−1^, indicate clearly that the Polish survey did not identify any soils highly enriched in Sb. However, we supposed that local cases of soil contamination with Sb are quite likely, particularly in the areas of present or historical Sb mining and processing. Such cases of soil enrichment in Sb were reported from various sites in the world that involved both historical and currently operating mines and smelters. Additionally, coal combustion, wastes dumps, power plants, battery factories, production and use of bullets, flame retardants and PETs, and automotive production can act as considerable sources of Sb in the environment (Filella et al. 2002; Reimann et al. 2010; Wilson et al. 2010; Lewińska et al. 2017; Földi et al. 2018; Li et al. 2018). A brief review of related bibliography indicates that mining and ore processing are the main sources of local soil enrichment in Sb. For instance, in the areas of mining activity in China (region Xikuangshan in Hunan), the content of Sb in soils reached 5045 mg kg^−1^ (He 2007) and the concentrations of Sb in waste material were even higher, i.e., 11,798 mg kg^−1^. Similar concentrations of Sb, up to 11,560 mg kg^−1^, were found in mine tailings in the French Massif Central (Courtin-Nomade et al. 2012). Very high concentrations of Sb in soils, over 9000 mg kg^−1^, were also reported from mine sites in Dúbrava, Slovakia (Hiller et al. 2012), and the data up to 4400 mg kg^−1^ Sb were recorded from Su Suergiu in Sardinia, Italy (Cidu et al. 2014). Macgregor et al. (2015) identified the sites with soil Sb concentrations up to 222 mg kg^−1^ in the surroundings of Glendinning mine (Scotland). Smelting activity can also cause soil contamination with Sb. Wilson et al. (2004) reported as high value of Sb concentration in soil as 80,200 mg kg^−1^ in the close neighborhood of a smelter in New Zealand. Another kind of industrial activities that can considerably enrich soils in Sb is battery factories. The concentrations of Sb up to 112 mg kg^−1^ were recorded from the surrounding of Pb-Sb recycling factory in Spain (Mykolenko et al. 2018). As antimony is widely used in the production of bullets that usually contain 2–5% of Sb (Johnson et al. 2005; Laporte-Saumure et al. 2011), shooting range soils are typically the sites reported as highly contaminated with Sb. In an Australian study, the concentrations of Sb in such soils reached 252 mg kg^−1^ (Sanderson et al. 2012), and the maximum soil concentration reported from the German Army shooting ranges was 437 mg kg^−1^ Sb (Spuller et al. 2007). A shooting range in Lucerna (Switzerland) was reported to contain extremely high concentration of Sb, i.e., 8230 mg kg^−1^ (Robinson et al. 2008), while considerably lower data were recorded from Norway, with the maximum value of 12 mg kg^−1^ Sb (Okkenhaug et al. 2016). Literature reported also high concentrations of Sb in soils in the neighborhoods of landfills and waste incinerators (Filella et al. 2009; Kabata-Pendias 2011). Hu et al. (2015) found 9000 mg kg^−1^ Sb in the top soils of brake pad dumpsite located in Miyun County in China. Okkenhaug et al. (2015) stressed that very high Sb concentrations can be present in waste electrical and electronic equipment, in vehicle plastic (1238–1715 mg kg^−1^), and in vehicle fluff (34–4565 mg kg^−1^). Although the latter data refer to solid wastes, and not to soils, high potential risk posed on the environment by the wastes rich in Sb disposed in the landfills cannot be ignored. Moreover, it was proven that the leachates from municipal solid wastes can contain high concentrations of Sb, up to 3.19 mg L^−1^ Sb (El-Fadel et al. 2002), that can easily move into soils and groundwater.

As pointed out above, the knowledge about Sb content in Polish soils is very limited. If considering the fact that Sb-rich ores were mined in the past in several sites of the Sudetenland, local enrichment of the environment in Sb can there be expected. Our preliminary study (Lewińska et al. 2017), carried out in the surroundings of the only historical stibnite (antimonite) mine in the Polish part of the Sudetes (Mączka and Stysz 2008), confirmed that mine dump material and soils there contain up to 427 mg kg^−1^ Sb. Elevated concentrations of Sb in soils should also be expected in the sites of former arsenic and polymetallic ore mining and processing, such as Złoty Stok, Czarnów, and Radzimowice (Karczewska et al. 2007, 2013; Krysiak and Karczewska 2007; Kabała 2015), as Sb occurs in those ores as an accompanying element (Mikulski 2010; Wilson et al. 2013; Macgregor et al. 2015). The soils of two Lower Silesian shooting ranges, in Wrocław and Oleśnica, turned out to contain enhanced concentrations of Sb, as it was revealed in our introductory study (Lewińska et al. 2017). The main aim of the present work was to deliver a possibly comprehensive picture of soil enrichment in Sb in Lower Silesia (SW Poland). It is worth mentioning that Polish law does not regulate the permissible concentrations of Sb in soils. Similarly, in most countries, Sb is absent in soil quality standards. However, the Geological Survey of Norway proposed a value 40 mg kg^−1^ as a permissible content of Sb in soils (Okkenhaug et al. 2016), and the Dutch soil standards accept the level of 100 mg kg^−1^ (van Leeuwen and Aldenberg 2012). Chang et al. (2002), the authors of a report for WHO, recommended the value 36 mg kg^−1^ as a maximum level of Sb in soils that can be fertilized with sewage sludge.

Various indices have been invented in order to classify the data on soil enrichment with potentially toxic elements, such as Sb. An enrichment factor (EF), proposed by Sutherland (2000), can be applied to assess the degree of pollution and the impact of anthropogenic activity onto soils (Kowalska et al. 2016; El-Badry and Khalifa 2017). EF is based on standardization of a tested element in relation to the reference one (Loska et al. 2004) and can be calculated according to the Formula (1). Sutherland (2000) proposed, accordingly, a five-category system for the assessment of soil pollution (Table 1).

1 $$ EF=\frac{\frac{\mathrm{Cn}\ \left(\mathrm{sample}\right)}{\mathrm{Cref}\ \left(\mathrm{sample}\right)}}{\frac{\mathrm{GBn}}{\mathrm{GBref}}} $$

where

Cn (sample): the content of a given element in the environment examined, e.g., in soil
Cref: the content of a reference element, e.g., Fe/Al/Ca/Ti/Sc/Mn, in the examined environment
GBn: the content of examined element in the reference environment
GBref: the content of the reference element, e.g., Fe/Al/Ca/Ti/Sc/Mn, in the reference environment

**Table 1:** Assessment of soil pollution based on the EF values (Sutherland 2000)

Value	Soil contamination category
EF < 2	Depletion to minimal enrichment suggestive of no or minimal pollution
2 < EF < 5	Moderate enrichment, suggestive of moderate pollution
5 < EF < 20	Significant enrichment, suggestive of significant pollution signal
20 < EF < 40	Very highly enriched, indicating a very strong pollution signal
EF > 40	Extremely enriched, indicating an extreme pollution signal

Another index, proposed originally as a quantitative measure of pollution in aquatic sediments (Müller 1986), and adopted to soils, is an index of geoaccumulation (*I*_geo_). It is based on the comparison of current concentrations of an element in soil or sediment and its geochemical background, calculated according to the following Formula (2):

2 $$ {I}_{\mathrm{geo}}={\log}_2\frac{\mathrm{Cn}}{1.5\times \mathrm{GB}} $$

where

Cn: concentration of metal (or metalloid) in the sediment or soil
GB: value of geochemical background related to local geological conditions
1.5: constant that allows to analyze natural variability of element in the environment

Accordingly, six classes of *I*_geo_ have been distinguished that define various levels of pollution (Table 2).

**Table 2:** The classes of soil quality based on *I*_geo_ values (Müller 1986; Loska et al. 2004; El-Badry and Khalifa 2017; Kowalska et al. 2018)

Class	Value	Soil quality
0	*I*_geo_ ≤ 0	Practically unpolluted
1	0 < *I*_geo_ < 1	Unpolluted to moderately polluted
2	1 < *I*_geo_ < 2	Moderately polluted
3	2 < *I*_geo_ < 3	Moderately to strongly polluted
4	3 < *I*_geo_ < 4	Strongly polluted
5	4 < *I*_geo_ < 5	Strongly to very strongly polluted
6	5 < *I*_geo_	Very strongly polluted

These two pollution indices can be used as useful tools to assess a potential environmental risk (Wang et al. 2018). It should be stressed, however, that they are both based on the total concentrations of toxic elements and do not take into account their mobility or solubility. On the other hand, however, it is obvious that the adverse effects posed on the environment by the presence of such elements depend not only on their total concentrations, but also on their speciation that determines a real and potential lability and bioavailability. The knowledge on speciation of potentially toxic elements is therefore of crucial importance from the standpoint of environmental risk. The methods commonly applied to determine operationally defined species of elements in soils are sequential extractions. They are believed to distinguish between the forms of elements that can be released from soil solid phase in various conditions. From among numerous procedures, the one optimized by Wenzel et al. (2001) was designed particularly for As, an element that behaves in soils similarly to Sb, and therefore, it is also the most appropriate for determination of operationally defined Sb species in soils (Müller et al. 2007; Ettler et al. 2010; Wilson et al. 2010; Fu et al. 2016; Okkenhaug et al. 2016). The procedure takes into account the anionic nature of metalloids and extracts their five fractions: (1) non-specifically sorbed, which can be considered “easily soluble”; (2) specifically sorbed; (3) associated with amorphous and poorly crystalline hydrous oxides of Fe and Al; (4) associated with well-crystallized hydrous oxides of Fe and Al; and (5) residual phases (Table 3).

**Table 3:** Procedure of sequential extraction based on Wenzel et al. (2001)

Fraction	Extractant	Extraction conditions	SSR*	Wash step
1	(NH_4_)_2_SO_4_ (0.05 M)	4 h shaking, 20 °C	1:25	–
2	(NH_4_)H_2_PO_4_ (0.05 M)	16 h shaking, 20 °C	1:25	–
3	NH_4_-oxalate buffer (0.2 M); pH 3.25	4 h shaking in the dark, 20 °C	1:25	NH_4_-oxalate (0.2 M); pH 3.25 SSR 1:12.5; 10 min shaking in the dark
4	NH_4_-oxalate buffer (0.2 M); + ascorbic acid (0.1 M) pH 3.25	30 min in a water basin at 96 ± 3 °C in the light	1:25	NH_4_-oxalate (0.2 M); pH 3.25 SSR 1:12.5; 10 min shaking in the dark
5	HCl:HNO_3_ (3:1)	Microwave digestion	1:50	

*SSR soil to solution ratio

The knowledge on speciation of a pollutant in soil is crucial for both assessment of environmental risk and planning a proper remediation strategy. Two opposite strategies of remediation may be applied to soils polluted with Sb: either its immobilization or decontamination. The latter approach may in some cases turn out unfeasible and may cause temporarily detrimental effects to soil biota. Moreover, the technologies of soil decontamination are very expensive. On the contrary, the techniques of immobilization are widely used because of their low costs, low risk of side effects, and social acceptance (Lewińska et al. 2018). Different kinds of amendments can be used for immobilization of Sb in soils, including organic compounds; oxides and hydroxides of Fe, Mn, and As; and different kinds of waste rich in those components (Leuz et al. 2006; Steely et al. 2007; Okkenhaug et al. 2016). However, in the areas contaminated by various metals and metalloids, immobilization can be challenging due to the differences in their mobility with changing pH (Barker et al. 2019; Okkenhaug et al. 2011, 2016).

The aim of this study was to recognize the sites in south-western Poland where soils are considerably enriched in Sb. For those sites, the pollution indices were calculated in order to roughly assess related contamination classes. In the cases of particularly high enrichment, potential solubility of Sb in soils was determined based on sequential extraction as an introductory step for the assessment of environmental risk.

## Materials and methods

### Soil material

**Fig. 1:**
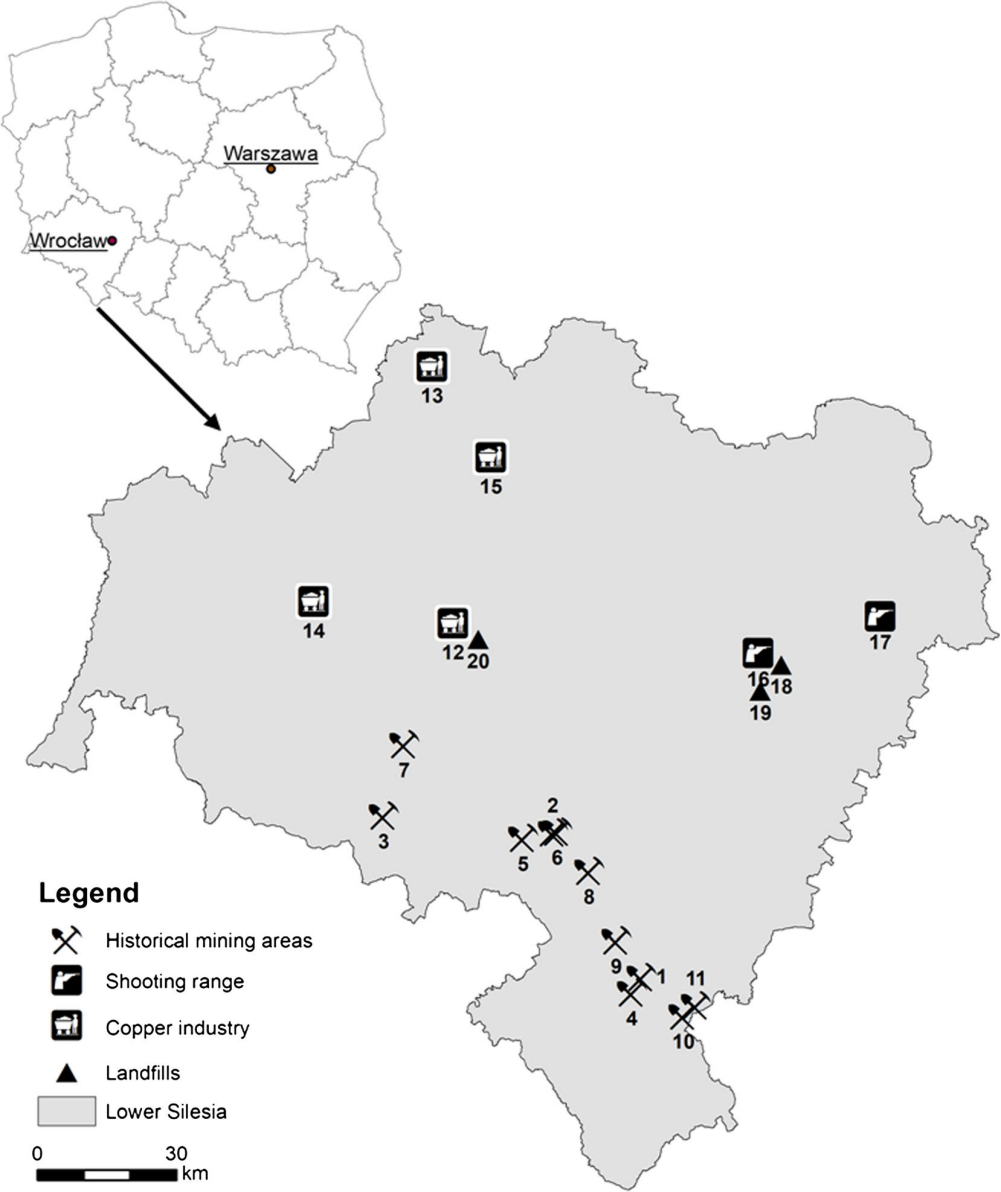
Location of sampling sites. Historical mining areas: 1. Bardo, 2. Bystrzyca Górna, 3. Czarnów, 4. Dębowina, 5. Dziećmorowice, 6. Modliszów, 7. Radzimowice, 8. Rościszów, 9. Srebrna Góra, 10. Złoty Stok, 11. Złoty Stok valley. Copper industry: 12. Legnica copper smelter, 13. Głogów copper smelter, 14. Wartowice tailings impoundment, 15. Żelazny Most tailings impoundment. Shooting ranges: 16. Wrocław, 17. Oleśnica. Landfills: 18. Swojec, 19. Ziębicka, 20. Nowa Wieś Legnicka

One hundred forty-four samples were collected from 20 locations (Fig. 1) in Lower Silesia, the south-western part of Poland, chosen as potentially enriched in Sb. They represented the surroundings of historical mining sites situated in various regions of Central Sudetes: a stibnite mine (Dębowina), arsenic mines (Czarnów, Złoty Stok) and polymetallic ore mines (Bardo, Bystrzyca Górna, Dziećmorowice, Modliszów, Radzimowice, Rościszów, Srebrna Góra) (Karczewska et al. 2007; Stysz et al. 2012; Mączka and Stysz 2013; Nejbert et al. 2013; Zagożdżon and Madziarz 2015; Lewińska et al. 2017), close vicinities of operating copper smelters in Głogów and Legnica (Karczewska 1996; Szerszeń et al. 1993), tailings impoundments Żelazny Most and Wartowice working for copper industry (Karczewska et al. 2017), currently operating and historical shooting ranges situated in Wrocław and Oleśnica (Lewińska et al. 2017), two closed municipal landfills in Wrocław, and a waste dumping site in Nowa Wieś Legnicka (Table 4). The landfill Ziębicka in Wrocław used, additionally, to serve as a dumping site for railway and gasworks, the operators that produced wastes rich in scrap-metal. The soil samples were basically collected from a surface soil layer (0–10 cm) and occasionally also from the subsurface layer (10–25 cm). The stones and coarse gravel particles (> 5 mm) were sieved out on site. Their approximate percentage was determined in the field.

**Table 4:** Description of sampling sites with their current type of use

Location	Sampling site	Geographical region	Depth (cm)	Number of samples	Type of use	Additional information
Historical mining areas	Bardo	Bardzkie Mts.	0–10	3	Dump	Very coarse and gravel
	Bystrzyca Górna	Sowie Mts.	0–10	6	Forest/dump	Coarse; up to 6070 mg kg^−1^ Pb, 6840 mg kg^−1^ Zn
	Czarnów	Rudawy Janowickie Mts.	0–10	4	Slag dump	Very coarse and gravel; up to 49,900 mg kg^−1^ As
			0–10	7	Forest/dump	
			0–10	4	Grassland	
			10–25	3	Grassland	
	Dębowina	Bardzkie Mts.	0–10	10	Forest/dump	Up to 2797 mg kg^−1^ As
			10–25	2		
	Dziećmorowice	Sowie Mts.	0–10	4	Woodland/dump	Up to 8899 mg kg^−1^ Ba
	Modliszów	Sowie Mts.	0–10	2	Forest/dump	Gravel; up to 14,460 mg kg^−1^ Pb
	Radzimowice	Kaczawskie Mts.	0–10	10	Forest/dump	Up to 27,500 mg kg^−1^ As
	Radzimowice (Olszanka)		0–10	4	Forest (alluvium of mine draining streams)	Up to 16,000 mg kg^−1^ As
	Rościszów	Sowie Mts.	0–10	3	Forest/dump	Up to 3310 mg kg^−1^ Pb
	Srebrna Góra	Sowie Mts.	0–10	9	Forest/dump	Up to 57,300 mg kg^−1^ Pb
	Złoty Stok	Złote Mts.	0–10	10	Forest/dump	Up to 44,520 mg kg^−1^ As
	Złoty Stok		0–10	6	Slag dump	Up to 16,680 mg kg^−1^ As
	Złoty Stok Trująca valley		0–25	4	Grassland	Up to 3620 mg kg^−1^ As
Copper industry	Legnica copper smelter	The Legnica plain	0–15	5	Forest	Up to 5250 mg kg^−1^ Cu and 1125 mg kg^−1^ Pb
	Głogów copper smelter	Dalkowskie Hills	0–15	3	Forest	Up to 1300 mg kg^−1^ Cu
	Wartowice tailings impoundment	Bory Dolnośląskie	0–15	1	Tailings impoundment	Up to 2790 mg kg^−1^ Cu
	Żelazny Most tailings impoundment	Lubińska upland	0–15	4	Tailings impoundment	Up to 1974 mg kg^−1^ Cu
Shooting range	Wrocław	The Wroclaw plain	0–10	12	Grassland	Up to 29,540 mg kg^−1^ Pb
			10–25	8	Under grass	
	Oleśnica		0–15	5	Forest	Up to 1388 mg kg^−1^ Pb
Landfills	Swojczyce	Silesian plain	0–25	6	Farmland, grassland	Municipal
	Ziębicka	Silesian plain	0–25	8	Grassland, woodland	From the gas plant/municipal
	Nowa Wieś Legnicka	The Legnica plain	0–10	2	Grassland, woodland	Wastes with unknown origin

**Fig. 2:**
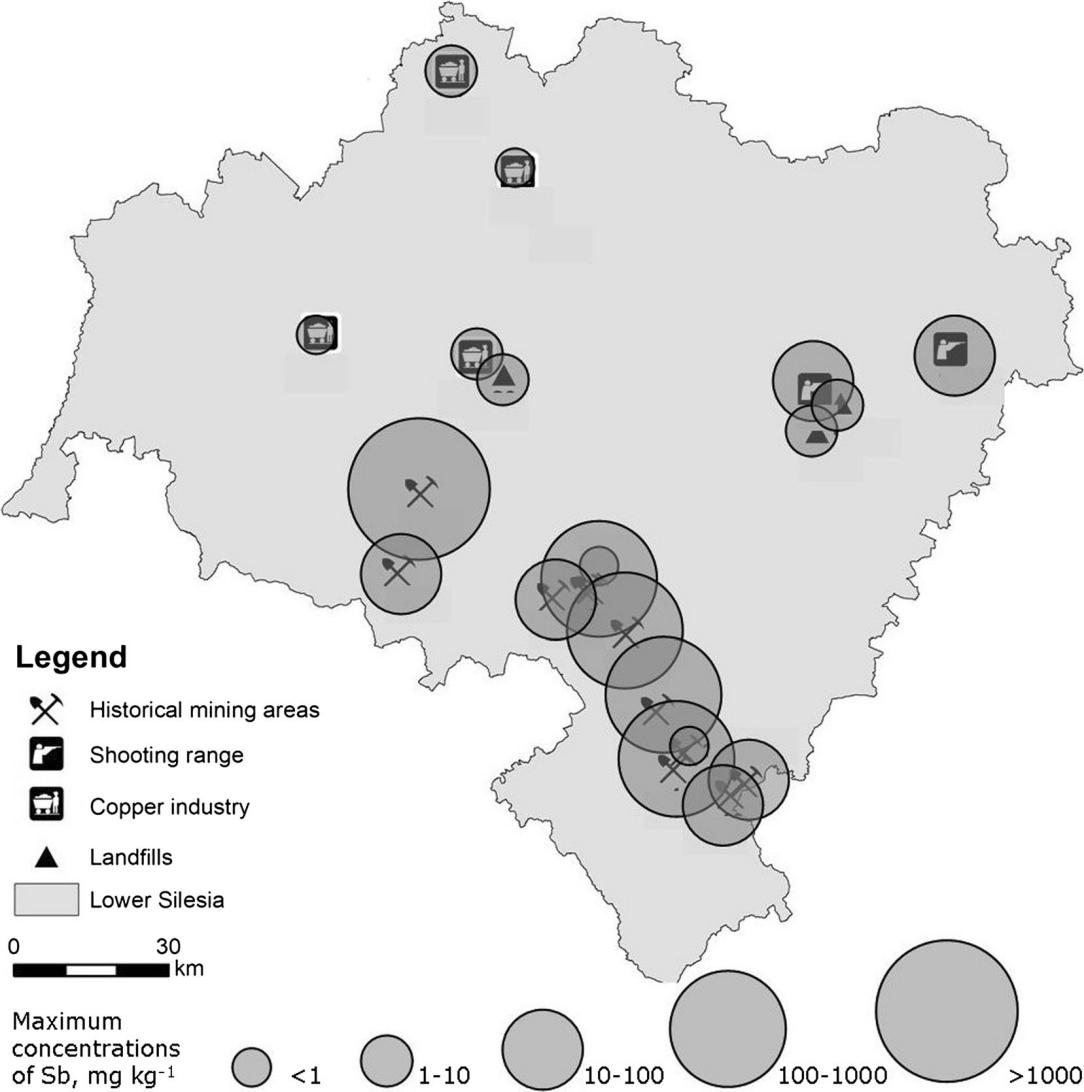
Maximum concentrations of Sb in soils

Air-dried soil samples were crushed and passed through a 2-mm stainless steel sieve. Basic soil properties were examined. Soil grain size distribution was determined using the hydrometer method (Gee and Bauder 1986), and the textural groups were assigned according to USDA classification (Soil Survey Staff 1999). The content of organic carbon (SOC) in soils was measured after sulfochromic oxidation on a tube digestion block, followed by titration with FeSO_4_ (ISO 14235 1998). Soil pH was determined potentiometrically in a suspension with 1 M KCl (*w*:*v* 1:1) by Seven Compact S220 (Mettler Toledo) with InLab Expert Pro-ISM combined electrode calibrated before the measurement with pH buffer solutions at pH 4.01 and 7.00. In selected samples, the mineralogical composition of the soil samples (clay fraction) was studied by X-ray diffraction (XRD), utilizing a ARL X’tra by Thermo Electron diffractometer. Pseudototal concentrations of Sb in soils (Fig. 2) were determined by ICP-MS (8800 Triple Quad, Agilent Technologies, Japan) after microwave digestion of samples in aqua regia (HNO_3_ + HCl, 1 + 3 suprapure, Merck), according to US EPA 3051 procedure (US EPA 2007). The same procedure was used for blanks and certified reference materials: fresh water sediment (CNS 392) and soils CRM027 and CRM052, provided by Sigma-Aldrich.

### Calculation of pollution indices

Two pollution indices: enrichment factor (EF) and geoaccumulation index (*I*_geo_), were calculated using the Formulas (1) and (2).

### Enrichment factor

For calculation of EF (Formula 1), iron was used as a reference element. Its pseudototal concentrations in all samples were determined by ICP-MS after microwave digestion of samples in aqua regia, as described above for Sb. These data were used as Cref values. The whole Earth crust was used as a reference environment, similarly as it was done by Sutherland (2000) and El-Badry and Khalifa (2017). Related concentrations of Sb and Fe in Earth crust (GBn and GBref) were set as 0.67 mg kg^−1^ and 5%, respectively (Kabata-Pendias 2011). Based on EF values, five categories of soil contamination with Sb were distinguished (Table 1).

### Geoaccumulation index

For calculation of *I*_geo_ (Formula 2), various values of Sb geochemical background (GB) were used for soils that differed in texture, in order to meet the requirement of considering local or specific geological conditions. Accordingly, the values of GB were set as (1) sands—0.19 mg kg^−1^ Sb, (2) medium-textured soils (loams and silts)—0.45 mg kg^−1^ Sb, and (3) heavy loams and clays—0.6 mg kg^−1^ Sb (Kabata-Pendias and Pendias 1999; Kabata-Pendias 2011).

### Sequential extraction procedure

Nine samples, relatively rich in Sb and also few with lower content of Sb and representative for historical mining sites in Bystrzyca Górna, Dębowina, Dziećmorowice, Srebrna Góra, Złoty Stok, and Radzimowice, as well as three samples of shooting range soils from Wrocław and Oleśnica (Table 5) were selected for sequential extraction. The procedure (Wenzel et al. 2001) involves five extraction steps and additional washing that follows extraction in the steps 3 and 4 (Table 3).

**Table 5:** Soil samples selected for sequential extraction

Location	Sampling site	Symbol	Textural group^a^	C org. %	pH	Total Sb mg kg^−1^
Historical mining sites	Dębowina (0–10 cm)	D-1	LS	17.4	3.2	179
	Dębowina (10–25 cm)	D-2	SL	4.0	3.4	437
	Bystrzyca Górna	B	LS	2.6	5.4	92.3
	Dziećmorowice	DZ	SL	5.2	5.6	151
	Srebrna Góra	SG	L	1.6	5.3	170
	Radzimowice	R-1	L	1.9	2.8	148
	Radzimowice (Olszanka)	R-2	L	3.6	7.1	5650
	Złoty Stok	ZS-1	LS	0.8	7.3	41.0
	Złoty Stok valley	ZS-2	SL	4.7	6.0	29.5
Shooting ranges	Wrocław	W	S	3.5	6.5	89.6
	Oleśnica 1	O1	S	0.1	8.0	4.15
	Oleśnica 4	O4	S	0.2	7.7	40.1

^a^*S* sand, *LS* loamy sand, *SL* sandy loam, *L* loam, *SiL* silt loam

After each extraction step, as well as after wash steps, the tubes were centrifuged for 15 min at 1700×*g*. Extracts were filtered through 0.45 μm cellulose acetate filter paper and immediately analyzed by ICP-MS (8800 Triple Quad, Agilent Technologies, Japan) for the concentrations of Sb. Blanks were performed in the same procedure as soil samples. All extractions were performed in duplicate. Recovery of the SEP, calculated as differences between “near total” concentrations of Sb (determined after aqua regia digestion of soil) and the sums of all fractions, ranged between 89.3 and 109.2%.

## Results and discussion

### Soil properties and total concentrations of Sb

#### Historical mining sites

Soils collected in the mine dump sites contained high contribution (45–80%) of skeletal fractions (> 5 mm), as roughly determined in the field (Karczewska et al. 2007, 2018, 2019). The analysis of grain size distribution (in the < 2 mm fraction) performed in the laboratory showed that the dominant textural group in the earthy fraction of soil samples collected from the mine sites was sandy loam (Table 6). Those soils were considerably rich in organic matter. The maximum content of organic carbon was 17.4% (in Dębowina), and the mean value for all mine site soils examined was 3.2%. Soil pH values ranged from 3.0 to 8.3, a median pH was 4.1, and most samples were assessed as strongly acidic. Such a low pH can be explained by the presence of sulfide minerals in the gangue rocks disposed on the dumps and their oxidation under propitious climatic factors. The mineralogical analysis (XRD) performed on selected samples showed that the main minerals present in clay fraction were quartz and phyllosilicates (kaolinite, illite, and chlorite). Additionally, gypsum and jarosite were found in the samples from Złoty Stok and Radzimowice, while iron oxides were highly abundant in Dębowina, Srebrna Góra, and Dziećmorowice. It should be noted that sulphides, mainly arsenopiryte, were identified in some samples (Złoty Stok and Dębowina), but none of Sb minerals were found. This effect can be attributed to limitations of XRD method that does not allow to detect mineralogical phases present in the amounts below 1%. Therefore, further, more advanced mineralogical analyses will be needed.

**Table 6:** Description of basic properties of soil samples and results of enrichment factor (EF) and geoaccumulation index (*I*_geo_) for antimony in soil samples

Location	Sampling site	Textural group^a^	C org. %	pH range	Sb concentrations, mg kg^−1^				EF			*I*_geo_		
					Min	Max	Mean	Median	Min	Max	Median	Max	Min	Median
Historical mining areas	Bardo	LS-SL	2.1–4.7	3.9–6.6	0.14	0.76	0.36	0.18	0.07	0.77	0.09	− 1.05	− 0.58	− 0.65
	Bystrzyca Górna	LS-SL	1.0–2.6	3.3–5.4	0.13	123	36.3	0.94	0.34	259	3.40	− 2.32	8.34	0.54
	Czarnów	S-SL	0.2–17.5	2.9–7.1	0.01	16.6	1.3	0.16	0.02	8.74	0.22	− 6.77	5.87	− 1.55
	Dębowina	S-L	0.5–17.4	3.0–6.0	0.33	437	54.7	0.60	0.30	823	0.82	− 1.34	9.34	0.01
	Dziećmorowice	LS-SL	2.3–5.2	7.5–8.0	0.05	151	62.8	50.0	0.09	209	64.8	− 4.14	7.39	2.39
	Modliszów	LS	0.8–6.0	4.4–5.5	0.08	99.2	49.6	49.6	0.42	317	177	− 1.76	8.44	4.85
	Radzimowice	S-SL	1.8–7.8	2.8–6.4	0.06	5650	499	13.3	0.10	2172	26.5	− 3.59	13.0	3.36
	Rościszów	LS-SL	1.2–5.2	3.7–4.8	0.001	0.09	0.03	0.01	0.00	0.09	0.01	− 9.28	− 1.73	− 7.23
	Srebrna Góra	LS-L	1.0–2.1	3.2–7.0	0.02	170	25.4	0.19	0.04	587	0.24	− 5.79	7.40	1.17
	Złoty Stok	S-SL	0.4–3.9	5.3–7.8	0.06	41.0	4.90	0.70	0.13	36.1	1.00	− 3.50	5.92	0.04
Copper industry	Legnica Cu smelter	LS-SiL	0.8–2.7	4.9–6.8	0.05	5.66	2.59	3.36	0.11	46.3	18.1	− 4.51	2.31	1.56
	Głogów Cu smelter	LS-SL	1.5–2.9	5.3–7.1	0.04	5.12	2.05	0.98	0.33	52.7	8.29	− 4.82	2.35	− 0.04
	Tailings impoundments	S-C	0.3–1.1	7.5–8.0	0.005	0.51	0.12	0.04	0.03	3.57	0.58	− 5.86	0.83	− 2.95
Shooting range	Wrocław	S-LS	0.1–5.8	5.6–7.2	0.14	93.4	17.0	4.16	0.93	578	28.4	− 0.98	8.36	3.35
	Oleśnica	S-LS	0.1–0.3	7.4–8.0	0.32	40.1	10.7	3.33	2.45	649	27.7	0.15	7.14	3.55
Landfills	Swojczyce	SL-L	1.4–3.2	5.1–6.8	0.03	1.47	0.33	0.12	0.12	4.56	0.12	− 4.70	1.12	− 2.55
	Ziębicka	LS-C	1.3–3.9	4.2–7.0	0.02	4.88	0.87	0.15	0.16	8.13	0.54	− 3.37	2.85	− 2.34
	Nowa Wieś Legnicka	S-SL	0.4–3.7	5.3–6.6	0.70	2.39	1.55	1.21	0.46	36.8	18.6	− 4.49	1.25	− 1.62

^a^*S* sand, *LS* loamy sand, *SL* sandy loam, *L* loam, *SiL* silt loam, *C* clay

**Fig. 3:**
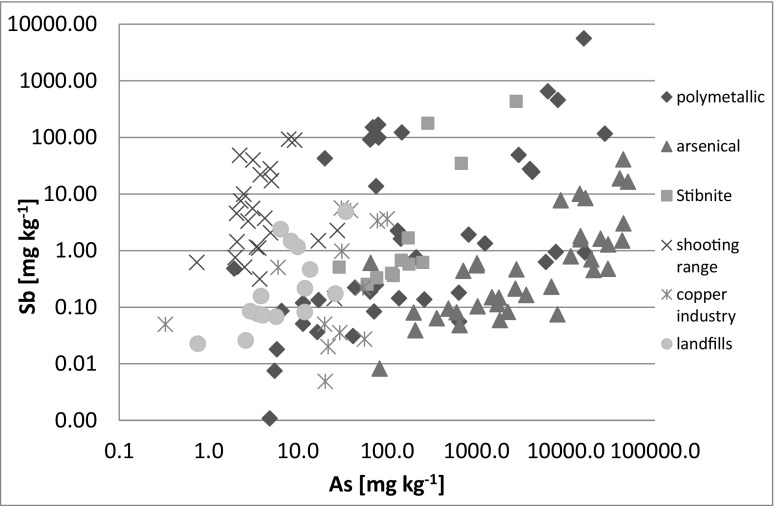
As and Sb co-occurrence in various types of deposits

The concentrations of Sb in soils of the mine sites differed considerably and ranged between 0.001 and 5650 mg kg^−1^. The lowest Sb concentrations were determined in Bardo and Rościszów. According to historical bibliographic sources, antimony ores accompanied by arsenic, copper, pyrite, and gold were acquired in those sites (Stysz et al. 2008, 2012), and the occurrence of Sb in waste dumps seemed very likely there; however, our survey showed that the waste rocks disposed in those sites were relatively poor in Sb. On the contrary, elevated concentrations of Sb were detected in soil samples collected from As mining sites in Czarnów and Złoty Stok, as well as from historical Ag and polymetallic ores mining sites in Srebrna Góra. Very high content of Sb in soils, over 100 mg kg^−1^, was also found in 10 samples collected from Dębowina, where stibnite was mined in eighteenth and nineteenth century, and in the samples that represented the zone of polymetallic mineralization in Bystrzyca Górna and Dziećmorowice. Quite unexpectedly, the maximum Sb concentration of all mine site samples, i.e., 5650 mg kg^−1^, was detected in alluvium of a stream supplied by mine drainage in the vicinity of polymetallic mine Radzimowice, and not in Dębowina. Moreover, very high concentrations of Sb were also found in mine dump soil samples collected in Radzimowice, which indicates that this area is not only extremely rich in As (Karczewska et al. 2007), the maximum soil concentration of which was determined as 192,500 mg kg^−1^ As (unpublished data), but also in Sb. The co-occurrence of As and Sb in soils was confirmed to be a typical feature for mine dump soils in Dębowina and Radzimowice (Fig. 3). Similarly high concentrations of Sb in soils of mine sites were recorded from many regions in the world where antimony was mined, for instance from Scotland (Macgregor et al. 2015), China (Wei et al. 2015), or Italy (Cidu et al. 2014). However, in none of those sites, Sb co-occurred with such extremely high concentrations of As. For instance, in the surroundings of the Xikuangshan Sb mine in China, the very high concentrations of Sb in soils (441–1472 mg kg^−1^) were accompanied by much lower content of As (32–354 mg kg^−1^) (Wei et al. 2015). Similarly, the samples from Sb mining sites in New South Wales in Australia contained 2735–4517 mg kg^−1^ Sb and 826–1606 mg kg^−1^ As (Wilson et al. 2013).

It will not be a revealing statement that the waste rock material of metal ore mine sites is usually highly heterogeneous, and consequently, the soils that develop on the dumps in a given mining site can differ dramatically in their composition. Consequently, the concentrations of Sb in soils developed on such dumps show large differentiation (Table 6), depending on the kinds of gangue rocks, their geochemistry, and mining techniques.

### Copper industry

The chemical properties of soil samples collected from copper tailings impoundments, their forelands, and from the surroundings of copper smelters differed strongly despite the fact that they had basically a common source of enrichment in metals and metalloids. The material collected from the Żelazny Most tailings impoundment had a sandy texture, while that from Wartowice represented clays. Also, the soils in the surroundings of two copper smelters showed a considerable diversity of their grain size distribution. The soils in the area of Głogów were relatively light, classified as sands and loamy sands, while in the surroundings of Legnica the dominant textural group of soils was silt loam (Table 6). The soil samples in this group were rather poor in organic carbon (1.5% on average), with the maximum content of 2.7% in a sample collected from the area of Legnica copper smelter, and negligible concentrations of organic matter in the tailings, particularly those from the Żelazny Most tailings impoundment. Soil pH values ranged from 4.9 to 8.0. Soils in the vicinity of copper smelters were predominantly acidic, while the pH of tailings was assessed as neutral to alkaline. High concentrations of Cu and Pb (Table 4) were a typical feature of all those soils, which indicated their enrichment in the elements originating from copper ores. The maximum concentrations of Cu and Pb in soils, i.e., 5254 mg kg^−1^ and 1125 mg kg^−1^, respectively, were recorded from the vicinity of the Legnica copper smelter. Similarly high concentrations were described by Cuske et al. (2017). The concentrations of Cu and Pb in tailings did not exceed 2800 mg kg^−1^ Cu and 630 mg kg^−1^ Pb. These data are comparable to those presented by Baran and Antonkiewicz (2017) and Karczewska et al. (2017). Notwithstanding a strong enrichment in Cu and Pb, the soils in the surroundings of copper smelters did not contain very high concentrations of Sb, with the maximum value of 5.66 mg kg^−1^ Sb in the sample collected in the neighborhood of Legnica and with an average value of 2.38 mg kg^−1^ (Table 6). Copper tailings were even poorer in Sb, with the maximum concentration 0.51 mg kg^−1^. Based on the presented results, it can be stated that mining and processing of copper ores in Poland do not make a considerable source of antimony in the environment.

### Shooting ranges

Two groups of shooting ranges examined in this study differed in their settings in a landscape. The shooting range Wrocław is located in the northern part of the city, in a close vicinity of residential buildings. The Oleśnica shooting range is surrounded by woodlands; however, the nearest houses are not very distant. This fact should be taken into account when assessing the environmental risk posed by soil pollution in those areas. Soil sampling sites were located mainly in the close proximities of firing points and backstops; however, the samples were also collected from other locations, throughout the entire areas of shooting ranges. All the samples had a sandy texture and were poor in organic carbon (Table 6). The average content of organic carbon in the shooting range soils was 1.6% and that in the samples from the Oleśnica shooting range was much lower, despite the fact that the facility has remained abandoned for over 30 years and is presently surrounded by woodlands. The pH of all soils in Wrocław was assessed as slightly acidic while that in the Oleśnica shooting range was neutral to alkaline, probably because of the occurrence of artifacts such as the pieces of concrete and bricks.

Much higher content of Sb in soils was recorded from the currently operating shooting range in Wrocław than in the soils of the historical shooting range. Particularly high soil concentrations of Sb were found in the area of backstop, with the maximum 93.4 mg kg^−1^. What should be emphasized, some samples collected from a subsurface soil layer contained also considerably high concentrations of Sb, up to 4.59 mg kg^−1^. The results obtained from our study are much lower than those reported from Switzerland, where shooting range soils contained even 8230 mg kg^−1^ Sb (Robinson et al. 2008), but they are higher than those found in Norway, where the maximum soil concentration of Sb was 12 mg kg^−1^ (Okkenhaug et al. 2016).

### Landfills

The samples were collected both from the surface layers of reclaimed landfills and from their surroundings. Three landfills examined in this study are surrounded by allotment gardens, arable fields, and wastelands. In the surrounding of the landfills Swojczyce and Nowa Wieś Legnicka, soil samples were collected from arable fields, while in the case of the Ziębicka landfill, the sampling sites were situated in its protection strip planted with trees as well as in allotment gardens and wastelands. The analysis of soil grain size distribution showed a variety of textural groups (Table 6). The concentrations of Sb in soils collected from the surroundings of landfills, as well as from their covering soil layers, were low, with the maximum 4.88 mg kg^−1^ Sb recorded from the Ziębicka landfill. The average concentration of Sb in soils of the Ziębicka landfill (0.87 mg kg^−1^) was higher compared to the Swojczyce landfill (0.33 mg kg^−1^), but lower than in Nowa Wieś Legnicka (1.21 mg kg^−1^). Such differences can be attributed to the kinds of wastes disposed in those sites. While the Swojczyce landfill collected almost exclusively municipal wastes, the Ziębicka landfill was additionally used for industrial purposes to dump the wastes produced by the railway operator and the gasworks. The facility in Nowa Wieś Legnicka was for a long time an illegal landfill where different wastes were dumped, very often of unknown origin. The diversity of Sb concentrations in landfill-affected soils can be ascribed to various properties of wastes. It should be stressed that the maximum values recorded in this study from such soils are considerably lower than those found in the surroundings of landfills where electronic wastes, plastics, and batteries were deposited (Gutiérrez- Gutiérrez et al. 2015; Okkenhaug et al. 2015; Mykolenko et al. 2018). On the other hand, however, the wastes dumped in well-protected disposal sites should not influence their surroundings. The concentrations of metals and metalloids in the soils neighboring with such landfills usually remain unaffected by their activity. For instance, the maximum concentrations of Sb in soils collected in the surroundings of the Seseña tire landfill in Spain, presented by Nadal et al. (2016), were as low as 0.06 mg kg^−1^.

To sum up an inventory part of this study, it should be stated that several locations in SW Poland turned out to have soils considerably enriched in Sb. It refers first of all to historical mining sites in Srebrna Góra, Dziećmorowice, Czarnów, Złoty Stok, and Radzimowice, as well as to the shooting ranges. The concentrations of Sb in those sites are usually highly heterogeneous, and the sites very rich in Sb are often spread in the areas slightly or moderately enriched. Therefore, it is particularly important to properly collect the representative soil samples with the purpose of environmental risk assessment. The maximum concentrations of Sb were found in Radzimowice, in the alluvium of a stream supplied with mine draining water, where Sb concentrations exceeded 5600 mg kg^−1^. The lowest concentrations of Sb, below 1.11 mg kg^−1^, were determined in the areas of landfills and their surroundings, in copper tailings and in two mine sites previously supposed to represent Sb-rich mineralization: Bardo and Rościszów. Those areas can be considered as uncontaminated by Sb.

The average concentration of Sb calculated for all soil samples in the studied areas was 21.8 mg kg^−1^ and was over 20 times higher than the mean Sb concentrations in Polish soils obtained from the monitoring networks (Pasieczna 2012; Kabata-Pendias 2011). It was also much higher than the average content of Sb in European soils provided by Salminen et al. (2005). The results show that the sites enriched in Sb have been rightly appointed for the purpose of general recognition. The study confirmed that there are various sites in Lower Silesia where soils contain relatively high concentrations of Sb. However, assuming that a permissible limit for total soil As was 40 mg kg^−1^, i.e., the value of the Norwich proposal (Okkenhaug et al. 2016), only 19 of all sites examined would be considered as polluted with Sb. Moving the limit to 100 mg kg^−1^, as in Dutch list (van Leeuwen and Aldenberg 2012), we could conclude that the real problem of undue concentrations of Sb in soils occurred in only nine of the sites examined.

### Indices of soil enrichment in Sb

#### Enrichment factor

The values of enrichment factor EF for Sb in all soil samples under study are presented in Table 6. The maximum EF values, up to 2172, were recorded from Radzimowice, the area extremely enriched in Sb and As. Very high factors were also obtained for Dębowina, up to 822, and for the soils of shooting ranges in Oleśnica and Wrocław, up to 649 and 578, respectively. The values of EF over 40 that should be classified as the extreme enrichment (Table 1) were also obtained for individual sites in Srebrna Góra (EF up to 587), Dziećmorowice (EF up to 209), Bystrzyca Górna (EF up to 167), and in the surroundings of Głogów copper smelter (EF = 53). Twenty-five samples were altogether classified as very highly and significantly enriched (EF 20–40). They were collected in the areas of Złoty Stok, Srebrna Góra, Modliszów, and Czarnów, as well as in both shooting ranges and in the neighborhoods of copper smelters. Eleven soil samples fell into the class of moderate enrichment (EF 2–5) that included several sites in the areas listed above, as well as the tailings in Żelazny Most and two landfills in Wrocław. Seventy-seven samples were classified as unpolluted, with EF < 2, though 50% of collected samples had the EF > 2 and were classified as contaminated, so they make a potential source of Sb in the environment.

### Geoaccumulation index

The values of *I*_geo_ are shown in Table 6. According to those values, the quality of 27 soil samples should be classified as extremely strongly polluted (class 6, *I*_geo_ > 5), with the maximum of *I*_geo_ = 13 in Radzimowice, in the alluvial soil affected by mine draining water. The other soil samples categorized in the 6th class of Sb geoaccumulation were those collected from historical mining sites in Dębowina, Srebrna Góra, Dziećmorowice, Bystrzyca Górna, and Złoty Stok, as well as in the shooting ranges. Five samples collected from shooting ranges were qualified to the class 5: strong to very strongly contaminated. Six samples that represented the shooting ranges and Czarnów mine site were qualified to the class 4: strongly contaminated, and further 14 samples, collected from the surroundings of copper smelters and one landfill in Wrocław—to the class 3: moderate to strongly polluted (2 < *I*_geo_ < 3). Based on *I*_geo_, 96 samples that made over 60% of the whole collection should be assessed as uncontaminated. It means, however, that nearly 40% of the samples bore the evident signs of an industrial, human-made impact.

Both indices, EF and *I*_geo_, showed high or very high soil pollution with Sb in the areas of historical mining activity as well as in shooting ranges. Those indices indicate a considerable environmental risk, as the values of EF > 20 and *I*_geo_ > 2 correspond with very high values of ecological risk index (Cai et al. 2015). It should be stressed, however, that a real scale of soil contamination in those areas is in fact not known. Soil enrichment with Sb, and with other potentially toxic elements, has most likely patchy or spotty patterns, which makes its assessment very difficult. Moreover, a release of Sb from soils is not monitored in any of the sites recognized as strongly contaminated.

### Sequential extraction

**Fig. 4:**
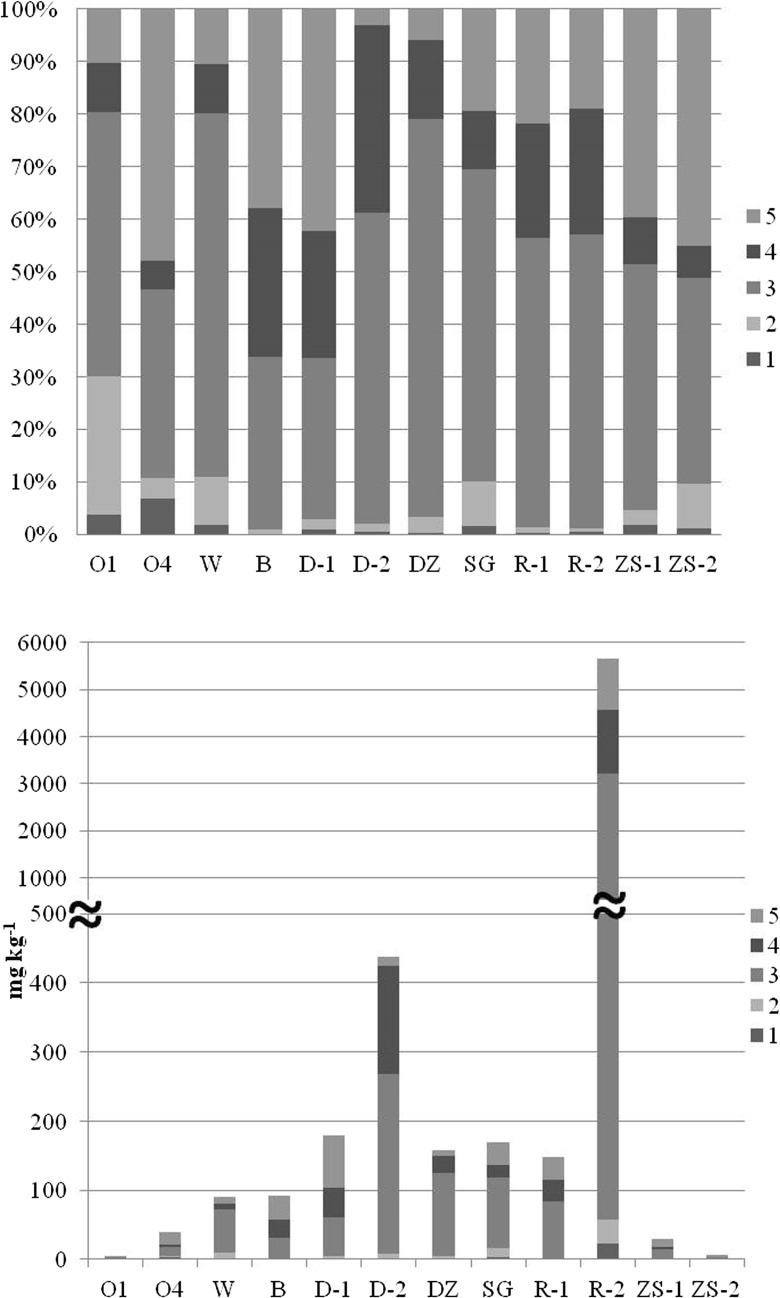
Sb speciation in soils determined in sequential extraction. Fractions: 1. non-specifically sorbed (easily soluble), 2. specifically sorbed, 3. bound to amorphous and poorly-crystalline hydrous oxides of Fe and Al, 4. bound to well-crystalline hydrous oxides of Fe and Al, 5. residual

The results of sequential extraction analysis, performed on 12 samples representative of the most enriched areas, are presented in Fig. 4. The sum of fractions 1 and 2 can be considered as easily mobilizable and therefore potentially dangerous to the environment. The highest percentage of potentially available Sb was found in a sample O1 collected from the Oleśnica shooting range, where the sum of fractions 1 and 2 reached 30% of total Sb, determined in aqua regia. This contribution should be assessed as extremely high compared to the results obtained by other researchers (Müller et al. 2007; Ettler et al. 2010). However, it should be stressed that the soil O1 was relatively poor in Sb and its total concentration in this soil was as low as 4.15 mg kg^−1^. Therefore, the absolute amount of Sb present in those two fraction, expressed in milligram per kilogram, remains in fact very low. Two other shooting range soils (W and O4) contained 10.9% and 10.6% of Sb in the fractions 1 and 2, respectively, which means that their sum made 9.8 and 4.3 mg kg^−1^, respectively. These values should be considered as relatively high. Müller et al. (2007) received almost 10% of sum first and second fraction by sequential extraction in samples collected in the area of historical copper mining and smelting in Harz Mountains in Germany. In our study, similar percentage of the fractions 1 + 2 (9.6–10.9%) was obtained from sequential extraction of Sb performed in the samples collected in Srebrna Góra (SG), Złoty Stok valley (ZS-2), and in Wrocław shooting range (W). Substantially lower (below 4.5%) was the potential solubility of Sb in the soils collected from the mine sites in Złoty Stok (ZS-1), Dębowina (D; both depths), and Dziećmorowice (DZ). Ettler et al. (2010) obtained quite comparable results from the sequential extraction of Sb in soils that represented the area of polymetallic ore mining and processing in Czech Republic. Those soils contained approximately 1.3–5.8% of Sb in the fractions 1 and 2. What is important for the assessment of environmental risk, the soils in Złoty Stok contain additionally high concentrations of As. The area is known as strongly polluted with arsenic, and numerous previous studies showed its high potential mobility (Krysiak and Karczewska 2007; Karczewska et al. 2007, 2013; Lewińska et al. 2018). The soils influenced by mining in Czarnów and Radzimowice (Karczewska et al. 2007) as well as those in Dębowina (unpublished data) are also considerably polluted with arsenic. It should be stressed therefore that potentially soluble fractions of both these elements will likely act together as environmental risk factors.

The lowest contributions of easily mobilizable Sb were found in mine dump soils R (both samples) and B, i.e., in the soils from Radzimowice (R-1 and R-2: 1.0 and 1.3%, respectively) and Bystrzyca Górna (1.1%), as well as in the samples collected from the site of former stibnite mine in Dębowina (D-1 and D-2: 2.8 and 1.7%, respectively). However, considerably low contributions of Sb in the fractions 1 and 2 (1%) in the sample R-2 made as much as 57.5 mg kg^−1^ of easily soluble Sb. In all the samples examined, independently of their origin, Sb was bound predominantly in the fraction 3, the content of which was in the range 37–75% of total Sb concentrations. This fraction, believed to be made mainly by Sb bound to amorphous Mn and Fe (hydro)oxides, should also be considered as potentially soluble, because it can be mobilized in reducing conditions. Similarly, the fraction 4, bound to crystalline oxides, that made in our soils from 6.2 to 38% of total Sb can be released to soil solution in anoxic environment. The lowest percentage of Sb in the fraction 4, below 9.5%, was found in the samples collected from shooting ranges as well as from the arsenic mine dump in Złoty Stok (ZS-1). The highest amounts of Sb bound to the crystalline oxides (fraction 4) were determined in the old mine dumps in Dębowina, Bystrzyca Górna, and Radzimowice. Several studies confirmed that Sb, similarly to As, may be released from soils through reductive dissolution of Mn and Fe (hydro)oxides under waterlogged conditions (Wilson et al. 2010; Tandy et al. 2018). Such processes would not, however, take place in most of the soils identified as enriched in Sb in this study, as they are not at risk of flooding, except for the alluvial soils in Radzimowice and the valley in Złoty Stok. Moreover, Sb released from the soil solid phase in such conditions would likely be resorbed on undissolved or on re-precipitating Fe hydroxides, due to the fact that a reduced form of Sb, i.e., Sb(III), is usually much more strongly sorbed than Sb(V) (Leuz et al. 2006; Mitsunobu et al. 2006; Filella et al. 2009; Wilson et al. 2010).

## Conclusions

This study was designed mainly as a screening based on examination of various sites selected as likely enriched in Sb. The results confirmed that there are several localities in Lower Silesia and the Sudetenland where soils contain elevated or even high concentrations of Sb. Those localities represented first or all the historical mine sites in which As, Sb, or polymetallic ores were acquired, as well as military shooting ranges. The study did not involve any advanced examination of a spatial distribution of soil enrichment with Sb or a real extent of pollution, but it indicated the sites where soil Sb concentrations and pollution indices were particularly high. Those sites should be examined more thoroughly in order to properly assess a related environmental risk. High percentage of Sb that was extracted from some samples in the fractions 1 and 2 of sequential extraction procedure provides a strong premise for further risk- and remediation-oriented examination of those soils. It refers particularly to the shooting range soils, the mine sites in Radzimowice, and Srebrna Góra, with special focus on alluvial soils in Radzimowice and the valley affected by arsenic mine tailings in Złoty Stok. A comprehensive assessment of environmental risk will be particularly important and should be urgently made in the case of shooting range soils, in which the enrichment in Sb is accompanied by very high contamination with Pb, as well as for alluvial soils highly polluted by As and prone to waterlogging. Based on pollution indices, attention should also be paid to the areas affected by historical mining in Dziećmorowice, Dębowina, and Modliszów. Those sites should be included into more advanced study aimed to determine the scale of soil contamination and to choose the most appropriate methods of remediation, considering first of all those based on immobilization of Sb and heavy metals using iron compounds. On the other hand, we can conclude that the tailings disposed in copper industry–associated impoundments do not indicate any particular enrichment in Sb. Furthermore, the extent of soil pollution with Sb in the surroundings of copper smelters and three Lower Silesian landfills can be assessed as very low and negligible from the standpoint of potential environmental risk.
